# 5-FU-hydrogel inhibits colorectal peritoneal carcinomatosis and tumor growth in mice

**DOI:** 10.1186/1471-2407-10-402

**Published:** 2010-08-02

**Authors:** Yongsheng Wang, Changyang Gong, Li Yang, Qinjie Wu, Shuai Shi, Huashan Shi, Zhiyong Qian, Yuquan Wei

**Affiliations:** 1 State Key Laboratory of Biotherapy and Cancer Center, West China Hospital, West China Medical School, Sichuan University, China

## Abstract

**Background:**

Colorectal peritoneal carcinomatosis (CRPC) is a common form of systemic metastasis of intra-abdominal cancers. Intraperitoneal chemotherapy is a preferable option for colorectal cancer. Here we reported that a new system, 5-FU-loaded hydrogel system, can improve the therapeutic effects of intraperitoneal chemotherapy.

**Methods:**

A biodegradable PEG-PCL-PEG (PECE) triblock copolymer was successfully synthesized. The biodegradable and temperature sensitive hydrogel was developed to load 5-FU. Methylene blue-loaded hydrogel were also developed for visible observation of the drug release. The effects and toxicity of the 5-FU-hydrogel system were evaluated in a murine CRPC model.

**Results:**

The hydrogel system is an injectable flowing solution at ambient temperature and forms a non-flowing gel depot at physiological temperature. 5-FU-hydrogel was subsequently injected into abdominal cavity in mice with CT26 cancer cells peritoneal dissemination. The results showed that the hydrogel delivery system prolonged the release of methylene blue; the 5-FU-hydrogel significantly inhibited the peritoneal dissemination and growth of CT26 cells. Furthermore, intraperitoneal administration of the 5-FU-hydrogel was well tolerated and showed less hematologic toxicity.

**Conclusions:**

Our data indicate that the 5-FU-hydrogel system can be considered as a new strategy for peritoneal carcinomatosis, and the hydrogel may provide a potential delivery system to load different chemotherapeutic drugs for peritoneal carcinomatosis of cancers.

## Background

Peritoneal carcinomatosis (PC) is one of the most common causes of incurability of intra-abdominal cancers, among which colorectal peritoneal carcinomatosis (CRPC) is a result of transcoelomic invasion by the primary cancer or intraperitoneal seeding during surgical manipulation. Approximately 8% of patients have isolated peritoneal seeding at the time of primary surgery, and 25% of patients with recurrence have disease confined to the peritoneal cavity [1,2]. Neither systemic chemotherapy nor intraperitoneal chemotherapy alone had any significant impact on survival [3]. The current goal of treatment for most abdominal/peritoneal metastases is palliative, rather than curative. It is imminent to sought new strategies to improve the therapeutic effects of intraperitoneal chemotherapy. Intraperitoneal infusion of 5-fluorouracil (5-FU) is frequently used for PC in patients with gastrointestinal malignancies and show positive results [3]. As an antimetabolite drug, 5-FU can incorporates into the DNA causing chain termination. To obtain maximal therapeutic effects, the prolonged and continuous administration is required.

Over the past decades, stimuli-sensitive polymers have attracted much attention due to their great biodegradability, biocompatibility, and responsiveness to the physical-chemical stimulus, such as temperature, pH and electrolyte composition [4-6], among which thermosensitive hydrogel has been extensively studied for its potential biomedical applications, including *in situ *gel-forming controlled drug delivery, cell encapsulation, and tissue repair [7-9]. Since a series of block copolymers consisting of PEG and PCL was first prepared, these triblock copolymers have been widely studied [10-12]. In our previous contribution, we have successfully prepared a series of thermosensitive hydrogels based on PECE copolymer [13,14], which are injectable flowing solutions at low temperature and undergo sol-gel transition at around body temperature. Besides, intrapleural, intraperitoneal and subcutaneous administration of PECE hydrogel were proved to be safe in BALB/c mice *in vivo *by acute toxicity test [15]. Therefore, this work is very important for the further application of PECE hydrogel as an injectable *in situ *gel-forming controlled drug delivery system.

In this study, we developed a 5-FU-loaded PECE hydrogel system (5-FU-hydrogel). The therapeutic effects of this system were evaluated in a murine CRCP model. The results presented here are positive and suggestive for a promising clinical application.

## Methods

### Synthesis and characterization of PECE copolymer

ϵ-Caprolactone (ϵ-CL, Alfa Aesar, USA), poly(ethylene glycol) methyl ether (MPEG, Mn = 550, Aldrich, USA), stannous octoate (Sn(Oct)_2_, Sigma, USA) and hexamethylene diisocyanate (HMDI, Aldrich, USA) were used without any further purification. All the materials used in this article were analytic reagent (AR) grade, and used as received.

PECE copolymer was synthesized as described in our previous works. Briefly, PEG-PCL diblock copolymer was prepared by ring-opening copolymerization of ϵ-CL initiated by MPEG using stannous octoate as catalyst; PECE triblock copolymer was synthesized by coupling PEG-PCL diblock copolymer using HMDI as coupling agent.

The obtained PECE triblock copolymer was first dissolved in AR grade dichloromethane, and reprecipitated from filtrate using AR grade petroleum ether. Then the mixture was filtered and vacuum dried to constant weight at room temperature. The purified PECE copolymers were kept in desiccators before further use. The copolymer was denoted as PEG-PCL-PEG (Y-X-Y) where × and Y represented the number average molecular weight (M_n_) of PCL and PEG block respectively.

Fourier transforms infrared spectroscopy (FTIR, 200SXV Infrared Spectrophotometer, Nicolet, USA), nuclear magnetic resonance analysis (^1^H-NMR, Varian 400 spectrometer, Varian, USA), and gel permeation chromatography (GPC, Agilent 110 HPLC, USA) were used to characterize the chemical structure and macromolecular weight of prepared PECE copolymer.

### Preparation of blank or 5-FU loaded PECE hydrogel

The PECE copolymer was dissolved in normal saline (n.s.) solution at designated temperature at the concentration of 25wt% to form blank PECE hydrogel, and then the blank PECE hydrogel was kept at 4°C before use. 5-FU loaded PECE hydrogel was prepared by direct dissolution method. Calculated amount of 5-FU was added into PECE hydrogel (25wt%) at 4°C (sol state) to form injectable homogeneous sol. The prepared 5-FU loaded PECE hydrogel was kept at 4°C before use.

### In vitro drug release profile of PECE hydrogel

5-FU was used as a model drug to determine the *in vitro *drug release behavior from PECE hydrogel. Phosphate buffer solution (PBS, pH = 7.4) was used as release medium. A membraneless model was introduced to assay the release behavior of PECE hydrogel *in vitro*. In detail, 200 μl of 5-FU loaded PECE hydrogel (25wt% of hydrogel containing 0.5 or 1 mg of 5-FU respectively) were placed into 4 ml-EP tubes and allowed to gel in an incubator at 37°C for 12 h. Then, the gels were immersed in 1 ml of PBS (pH = 7.4) and were shaken at 100 rpm at 37°C. At specific time, all the release media were removed and replaced by pre-warmed fresh release media. After centrifugation at 13000 rpm for 10 min, the supernatant of the removed release media were collected and stored at -20°C until analysis. The concentration of 5-FU in the collected supernatants were determined by UV spectrophotometer at 286 nm. All the release study experiments were repeated three times. All data are expressed as the mean ± S.D.

### Cell and animals

Colon cancer cell line CT26 were obtained from ATCC (American Type Culture Collection) (Manassas, VA), and cultured in RPMI-1640 (Gibco), supplemented with 10% fetal bovine serum (FBS) plus ampicillin and streptomycin routinely in a 5% CO_2 _incubator at 37°C. Female Balb/c mice (6-8 weeks old) were purchased from the Laboratory Animal Center of Sichuan University. Animal experiments were approved by the Institutional Animal Care and Treatment Committee of Sichuan University (Chengdu, China).

### Observation of the release of methylene blue in methylene blue-loaded hydrogel in mice

Methylene blue-loaded hydrogel was prepared with the same way as 5-FU-hydrogel. The methylene blue-loaded hydrogel with a form of solution was infused into murine abdominal cavity. The changes of methylene blue were observed.

### RCPC Mice model and treat plan

RCPC mice model was established and subsequently treated. Briefly, 2 × 10^5 ^CT26 cells were injected into murine abdominal cavity. Five day later, these Balb/c mice were divided four groups (n = 10) received n.s., hydrogel, 5-FU (25 mg/kg) and 5-FU-hydrogel, respectively. All of the treatments were performed once per week for two weeks. Mice were sacrificed on 20^th ^day, the size and numbers of tumor nodes were measured.

### Evaluation of systemic toxicity

To investigate potential side effects or toxicity on mice during the treatment, they were observed continuously for relevant indexes such as weight loss, diarrhea, anorexia, skin ulceration and toxic deaths. The tissues of heart, liver, spleen, lung, kidney and brain were stained with H&E. The blood was obtained from tail vein once per week to detect the hematologic toxicity.

## Results

### Synthesis and characterization of PECE copolymer

The PEG-PCL diblock copolymer was prepared by ring-opening copolymerization of ϵ-CL initiated by MPEG using stannous octoate as catalyst, and biodegradable PECE triblock copolymer was synthesized from PEG-PCL diblock copolymer using HMDI as coupling agent. FTIR, ^1^H-NMR and GPC were used to characterize the chemical structure of PECE copolymer.

The M_n _and PEG/PCL block ratio of PECE triblock copolymer calculated from ^1^H-NMR spectra was 3408 and 960/2448 respectively. Molecular weight and polydispersity (PDI, Mw/Mn) of PECE triblock copolymer determined by GPC were 4391 and 1.30 respectively. FTIR, ^1^H-NMR, and GPC results indicated that the PECE triblock copolymer designed by controlling the feed composition were prepared successfully.

### Temperature-dependent sol-gel-sol transition behavior

**Figure 1:**
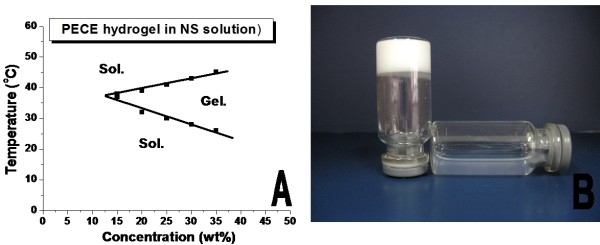
**Sol-gel-sol phase transition of PECE hydrogel**. A: Sol-gel-sol phase transition diagram. When the concentration was above corresponding critical gel concentration (CGC), the aqueous solution of PECE copolymer undergo a sol-gel-sol transition as the temperature increases.B: Photograph of PECE hydrogel at 10°C (right) and at 37°C (left). At low temperature, the hydrogel is an injectable flowing sol, and forms a non-flowing gel in body temperature.

As shown in Figure 1A, the PECE copolymer displayed a special temperature-dependent sol-gel-sol transition in n.s. When the concentration was above corresponding critical gelation concentration (CGC), the aqueous solution of PECE copolymer undergo a sol-gel-sol transition as the temperature increases. In Figure 1B, at low temperature, the hydrogel is an injectable flowing sol, and forms a non-flowing gel in body temperature.

### In vitro drug release profile

**Figure 2:**
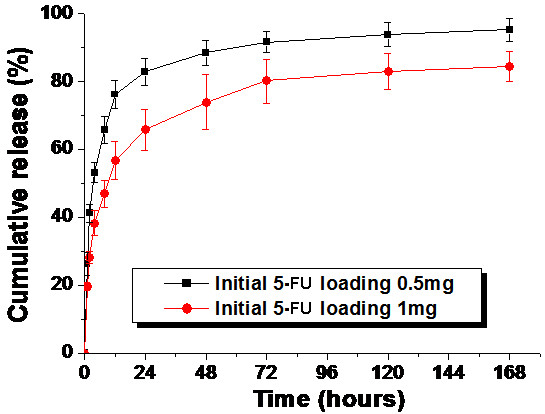
**In vitro release behavior of 5-FU from PECE hydrogel (25wt%)**. Error bars represent the standard deviation (n = 3). 5-FU could be released from PECE hydrogel in a sustained period. The initial drug loading amount had great effect on 5-FU release profile. With lower initial drug loading amount (black square), 5-FU released faster and reached higher cumulative release rate compared to higher initial drug loading hydrogel (black circle).

*In vitro *release behavior of 5-FU from PECE hydrogel was studied, and the results were shown in Figure 2. 5-FU could be released from 5-FU-hydrogel in a sustained period. The initial drug loading amount had great effect on 5-FU release profile. With lower initial drug loading amount (0.5 mg), 5-FU released faster and reached higher cumulative release rate (95.3%) compared to higher initial drug loading hydrogel (84.6%). In PECE hydrogel containing 0.5 mg 5-FU, an initial burst release of 26.2% of loaded 5-FU occurred in the first one hour, followed by releasing of 82.9% in one day, whereas, with twice amount of loaded 5-FU, the cumulative release rate of one hour and one day were 19.8% and 65.8% respectively.

### The hydrogel prolongs the release of methylene blue and enhances the permeability of peritoneum

**Figure 3:**
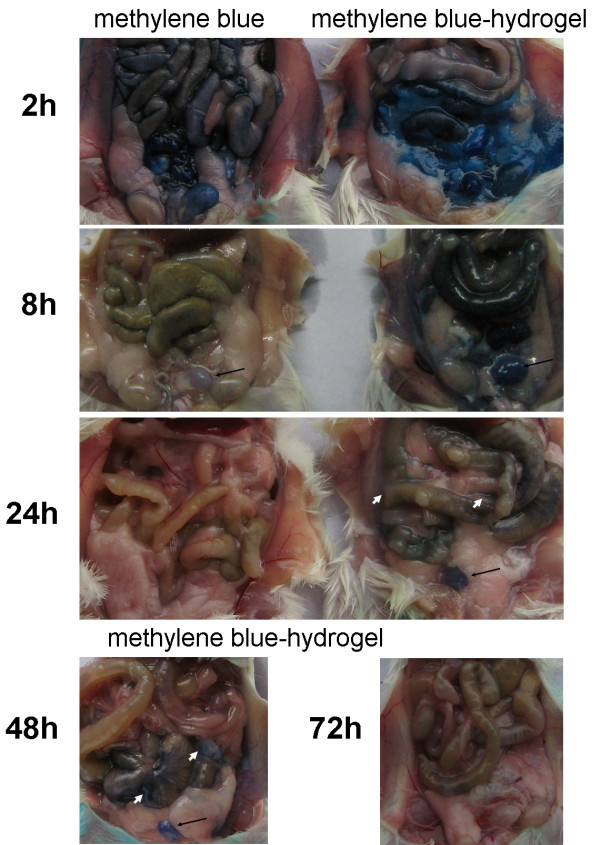
**The hydrogel system delayed release and increased uptake of methylene blue**. Mice were intraperitoneally injected methylene blue-hydrogel and then sacrificed at 2 h, 8 h, 24 h, 48 and 72 h to observe the release and increased uptake of methylene blue. The hydrogelization of methylene blue resulted in delayed release and increased uptake until 48 h (white short arrows), while methylene blue alone were discharged at about 8 h. The delayed release of methylene blue also observed in bladder (black long arrows).

To observe the release or uptake process of drug loaded in hydrogel in a way of intraperitoneal administration. The methylene blue-loaded hydrogel was infused into murine abdominal cavity for mimic the condition. The release of methylene blue was significantly delayed (Figure 3). Interestingly, methylene blue was observed in bladder lasting more than 48 hours. These findings suggest that the hydrogel system not only act as a sustained drug delivery system, but also improve the permeability of methylene blue into abdominal tissue, further into blood.

### The 5-FU-hydrogel significantly inhibits the peritoneal dissemination and growth of colorectal cancer

CRPC mice model was established and used to evaluate the therapeutic effects. To observe dose-dependent effects, we first performed a dose gradient assay in vivo. CRPC mice were divided four groups (n = 5) treated with 5-FU-hydrogel loaded different doses of 5-FU (25 mg/kg, 50 mg/kg, 100 mg/kg and 250 mg/kg). However, the inhibitory effects were not significantly improved when the dose of 5-FU-hydrogel increased more than 25 mg/kg (data not shown). Therefore, the dose of 5-FU at 25 mg/kg was selected for further study.

**Figure 4:**
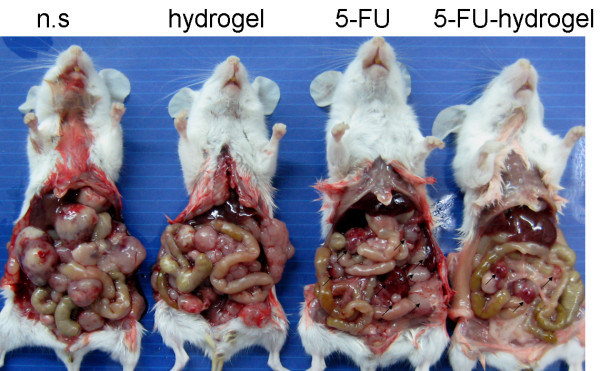
**5-FU-hydrogel system effectively inhibited the peritoneal dissemination and growth of colorectal cancer**. The decreased tumor nodes and tumor size were observed followed by intraperitoneal infusion of 5-FU-Hydrogel.

To investigate the therapeutic effects of 5-FU-hydrogel, forty CPRC mice were divided four groups treated with n.s, hydrogel alone, 5-FU alone and 5-FU-hydrogel. The results showed that the tumor node numbers from 5-FU-hydrogel-treated group were significantly less than that from other groups (table 1). Furthermore, the size of tumor nodes was significantly smaller in comparison to other groups (Figure 4). Subsequently, the increased tumor free rate and survival rate were also observed in 5-FU-hydrogel-treated group. This finding indicates 5-FU-hydrogel not only impair tumor dissemination, but also inhibits growth of implanted tumor.

**Table 1:** The enhanced anti-tumor effects of 5-FU-hydrogel

Groups	Mean number of tumor nodes	Tumor-free rate %	Survival rate %
n.s	20.2 ± 10.08	0	62.5
Hydrogel	23.67 ± 6.98	0	75
5-FU	11.6 ± 3.8	0	62.5
5-FU + Hydrogel	5.3 ± 4.04*	42.5**	100*

*,**Indicates p < 0.05 and p < 0.01, respectively

### Systemic toxicity analyses

**Figure 5:**
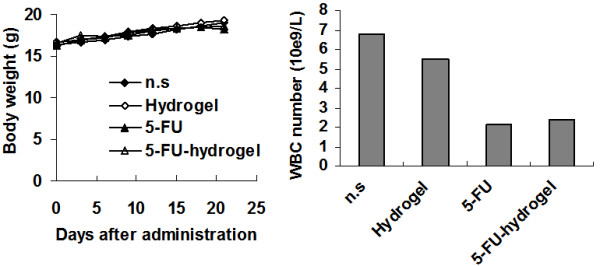
**Lack of toxicity-dependent weight loss in mice pretreated with 5-FU-hydrogel**. Body weights of tumor-bearing mice were plotted at 2-d intervals and the curve of the HNP1 group paralleled very closely to that of the control groups with no significant differences among them (A, P > 0.05). Mean ± SD. On the 14th day, the number (mean value) of white blood cells from 5-FU-hydrogel-treated mice was higher than that from the 5-FU-treated group.

In this study, we also investigated the cytotoxicity of the treatment. Body weights of mice were monitored every five days. No obvious signs of adverse results were observed in body weight, behavior or feeding. The serum ALT (alanine aminotransferase) and AST (aspartate aminotransferase) levels were among normal range, as well as creatine levels. In addition, hematoxylin and eosin staining sections of liver, kidney, and lung were observed under microscope. No organ hemorrhage was found (data not shown). However, the hematologic toxicity showed significant difference. On the 14^th ^day, the number of white cells from 5-FU-hydrogel-treated mice was higher than that from the 5-FU-treated group (Figure 5). The data indicate hydrogel system attenuate the hematologic toxicity of 5-FU in spite of the increased permeability.

## Discussion

The current therapy recommended by guideline of NCCN for CRPC is palliative. Nevertheless, the treatment of disseminated carcinomatosis with cytoreductive surgery (ie, peritoneal stripping surgery) and perioperative hyperthermic intraperitoneal chemotherapy has been considered as a new therapeutic concept to cure CRPC [16,17]. Importantly, intraperitoneal chemotherapy for CRPC is recommendable and should be immediately performed along with peritoneal surgery so that residual tumor cells do not get trapped in the postoperative fibrin adhesions [18]. In this study, we reported that the hydrogel system, a biodegradable poly (ethylene glycol)-poly (ϵ-caprolactone)-poly (ethylene glycol) (PEG-PCL-PEG, PECE) triblock copolymer, can be used as a delivery vector to load chemotherapeutic drugs for intraperitoneal infusion chemotherapy. Here, methylene blue was used to mimic the release and uptake of drugs; 5-FU was used as a model drug. Although the methylene blue and 5-FU may be released from the hydrogels at different rates, the results strongly support that PECE hydrogel can endue the delayed-release properties and increased permeability of drugs. For intraperitoneal chemotherapy, prolonged stay and increased permeability of drugs are helpful for better disease control. In fact, the 5-Fu-loaded-hydrogel system did show significantly increased therapeutic effects for CRPC.

In recent years, biodegradable thermosensitive hydrogels have been wildly investigated as controlled drug delivery system, in which pluronic F127 and PEG-PLGA copolymer were the most extensively studied ones [19,20]. Pluronic F127 is a commercial thermosensitive hydrogel, which has been widely used as emulsifiers, wetting agents, and solubilizers [20,21]. However, the critical micelle concentration (CMC) of Pluronic F127 is very high due to weak hydrophobicity of PPG block. Pluronic F127 forms a fast-eroding gel and can not persist longer than a few hours in vivo. Furthermore, Pluronic was found to induce the toxic enhancement of plasma cholesterol and triglycerol because it was non-biodegradable and could be accumulated in the body [22,23]. Thus, the wider application of Pluronic in many biomedical fields was greatly restricted.

In our work, we have synthesized a biodegradable Pluronic analog. Polycaprolactone (PCL) was used instead of PPG to synthesize PECE copolymer, and the formed hydrogel could persist approximately 5 days *in vivo *by intraperitoneal administration. Incorporation of biodegradable and strong hydrophobic block into Pluronic copolymer backbone will result in distinct decrease in macromolecular weight after degradation, easier elimination from the body, and evident decrease in CMC. Unlike PEG-PLGA hydrogel [19], the PECE hydrogel forms a soft gel at body temperature and could not hurt any organs in intraperitoneal cavity, which is suitable for intraperitoneal administration.

Currently, the drugs-combined intraperitoneal chemotherapy (ie. 5-FU and mitomycin-C) and intraperitoneal chemohyperthermia (IPCH) are recommended treatment modalities owing to improved therapeutic effects on PC [16,24,25]. Furthermore, the combination of IPCH and cytoreduction has shown curable potential to PC in some clinical trials [24,26,27]. However, recurrence is still a frequent event after optimal cytoreduction and IPCH for CRPC [28], and the modalities are still investigational and do not endorse extended applications outside of a clinical trial. Enhancing the effects of intraperitoneal infusion chemotherapy should be responsible for improve curable potential of the combined modalities to PC.

In the 5-FU-Hydrogel system, temperature sensitivity of Hydrogel is very important. At low temperature (<32°C), the fluidity of the system enable the easy infusion and extensive distribution. The murine body temperature is about 37°C. When the system was infused intraperitoneally, the formed gel phase guaranteed the delayed drug release, and increased drug uptake.

## Conclusions

This study demonstrates that the biodegradable PECE hydrogel is a promising *in situ *gel-forming controlled drug delivery system. Aqueous solution of PECE copolymers undergoes sol-gel-sol transition as temperature increases. *In vitro *drug release behavior indicated that PECE hydrogel could provide a sustained release profile.

## Competing interests

The authors declare that they have no competing interests.

## Authors' contributions

YS W and GY C Performed experiments, interpreted results, drafted manuscript. YS W and ZY Q Drafted manuscript, critical revision to manuscript, designed experiments, interpreted results. YQ W Drafted manuscript, critical revision to manuscript, designed experiments. L Y, QJ W and HS S designed and conducted experiments. All authors have read and approved the final manuscript.

## Pre-publication history

The pre-publication history for this paper can be accessed here:

http://www.biomedcentral.com/1471-2407/10/402/prepub
